# A consideration of what is meant by automaticity and better ways to measure it

**DOI:** 10.3389/fpsyg.2014.01537

**Published:** 2015-01-12

**Authors:** David A. Keatley, Derwin K. C. Chan, Kim Caudwell, Nikos L. D. Chatzisarantis, Martin S. Hagger

**Affiliations:** Health Psychology and Behavioural Medicine Group, School of Psychology and Speech Pathology, Curtin UniversityPerth, WA, Australia

**Keywords:** implicit, implicit association test, automaticity, methods, habit

Existing models of exercise behavior are insufficient in predicting outcomes, this point is shown by the relatively high levels of unexplained variance in exercise behavior in meta-analyses of social cognitive theories and models (Chatzisarantis et al., 2003; Hagger and Chatzisarantis, 2009). Researchers are beginning to recognize the importance of implicit, automatic processes in the prediction of health behaviors (Dimmock and Banting, 2009; Keatley et al., 2012, 2013b). The research by de Bruijn et al. (2014) is useful for highlighting the importance of automaticity in exercise behavior. We commend the authors on investigating an important approach to automaticity and exercise behavior. There were, however, some points with which we disagree. We think that the authors do not provide a clear account of what they mean by automaticity–an issue that is essential for the operationalization of the construct. Bargh (1994), for instance, suggested automaticity has four characteristics: awareness, intention, efficiency, and control; it is not clear whether de Bruijn and colleagues automaticity adheres to this. In particular, we contend that the explicit measure of automaticity used in their research is not an optimal way to assess implicit, impulsive processes. Furthermore, we contend that implicit measures, such as the implicit association test (IAT; Greenwald et al., 1998) would be better positioned as measures of non-conscious processes. The present commentary focuses on pre-behavior automatic associations, which we contend are better assessed by existing implicit measures, rather than during-behavior automatic “processes.”

Taking our perspective from existing implicit theories and models (Strack and Deutsch, 2004; Levesque et al., 2008), we suggest that the authors should clearly outline that their definition of automaticity reflects more conscious processes and self-reporting. De Bruijn and colleagues' proposal that automaticity is determined by the number of times a person has made a decision and goes through the processes does not necessarily imply automaticity. The experience of automaticity in de Bruijn and colleagues' study is self-reported and does not necessarily measure the reality of automaticity; people may report they do things automatically, but, in reality a lot of conscious effort goes into their actions (see Hagger et al., 2014).

## What are implicit processes?

Given the recent rise in research focusing on non-conscious, automatic, or implicit processes, clear definitions of what each researcher means by their terms is fundamental in reducing confusion between what is meant by each term and how to measure it (Payne and Gawronski, 2010). The explicit measure used in the de Bruijn et al.'s study does not appear to assess implicit or impulsive processes for either measurement type or psychological attribute. One way of defining and using the term “implicit” refers to the assessment of individuals' psychological attributes without the need for explicitly asking people (Fazio and Olson, 2003); de Bruijn and colleagues' automaticity measure is not consistent with this definition. Other researchers have defined automaticity as constructs or systems that are assessed by particular types of measures that do not require explicit, conscious introspection (Banaji, 2001). Instead, de Bruijn and colleagues' measure appears to be something of a meta-cognitive variable that reflects participant deliberations on the extent to which their exercise behavior is automatic. We suggest that the type of automaticity de Bruijn and colleagues referred to may actually be better measured by existing implicit measures, such as the IAT.

## Automaticity as associations

de Bruijn et al. outline that “repetition in stable contexts strengthens associations between the situational cue and the behavioural action” (p. 768). While we fully endorse this interpretation of existing findings in the literature, we suggest that existing implicit tests would be a better measure of these associations (Dimmock and Banting, 2009; Conroy et al., 2010). Implicit measures provide a clear indication of whether the associations between cue and action are influenced by automatic, implicit or deliberative processes. Measures of implicit constructs such as the IAT (Greenwald et al., 1998) or go no-go tasks (Nosek and Banaji, 2001), for example, that measure the speed of association between targets and categories would be more fit-for-purpose to tap automaticity as it is defined by de Bruijn and colleagues. Such tests could be adopted to measure the extent to which associations between contexts and behaviors (e.g., leisure time context and exercise behaviors) are automatic; this could be used concurrently with the measure of automaticity used by de Bruijn and colleagues' to provide some concurrent validity.

## Measurement issues

One pertinent issue in research involving implicit and explicit measures is measurement correspondence. In de Bruijn and colleagues' study, it may be that the explicit measure of automaticity is predicted by the explicit predictor variables due to the correspondence of measurement type. The current trend appears to be that implicit measures better predict spontaneous outcomes and behaviors than planned, self-reported behaviors (Perugini et al., 2010, 2011). Similarly, explicit, self-report measures seem better suited to predicting reflective, self-report behaviors. In de Bruijn and colleagues study, even though the explicit measure of automaticity was measured 2 weeks after the baseline assessment of past behavior and social cognitive variables, the study could not exclude the possibility that common method variance (Spector, 2006) and semantic similarity (Arnulf et al., 2014) inflated the associations between the study variables. It is important that future research should include implicit measures when examining the relationship between non-conscious processes and social cognitive variables. Furthermore, previous research has shown that objectively-measured behaviors (e.g., accelerometer for physical activity, free choice task for motivation) are better predicted by implicit measures (Keatley et al., 2013a). Future research should therefore incorporate alternative objectively-measured outcome variables, which may be better measured by implicit measures. However, it should be noted that implicit measures, such as the IAT, are not process-pure, they are subject to the influence of control processes and biases. There are, however, models (e.g., process dissociation analyses) that have been developed to “purify” the measure of automatic associations (e.g., Payne, 2001).

## Conclusion

While we support the research conducted by de Bruijn and colleagues, we contend that there are limitations in the conceptualization of automaticity and the results presented. Given the definition of automaticity outlined by the authors, existing measure of implicit associations appear to be better suited to measure automaticity. We therefore think the research would be improved by the inclusion of implicit measures. Furthermore, we encourage future research to include outcome variables of automaticity that may be better predicted by related implicit measures. Finally, researchers using longitudinal designs should be mindful to not allow mere measurement effects or measure correspondence or common method variance to affect results.

### Conflict of interest statement

The authors declare that the research was conducted in the absence of any commercial or financial relationships that could be construed as a potential conflict of interest.
